# Automated image analysis of instagram posts: Implications for risk perception and communication in public health using a case study of #HIV

**DOI:** 10.1371/journal.pone.0231155

**Published:** 2020-05-04

**Authors:** Alicia L. Nobles, Eric C. Leas, Seth Noar, Mark Dredze, Carl A. Latkin, Steffanie A. Strathdee, John W. Ayers

**Affiliations:** 1 Division of Infectious Diseases and Global Public Health, Department of Medicine, University of California San Diego, La Jolla, California, United States of America; 2 Division of Health Policy, Department of Family Medicine and Public Health, University of California San Diego, La Jolla, California, United States of America; 3 School of Media and Journalism, University of North Carolina at Chapel Hill, Chapel Hill, North Carolina, United States of America; 4 Department of Computer Science, Johns Hopkins University, Baltimore, Maryland, United States of America; 5 Department of Health, Behavior and Society, Johns Hopkins Bloomberg School of Public Health, Baltimore, Maryland, United States of America; University of Zurich, SWITZERLAND

## Abstract

People’s perceptions about health risks, including their risk of acquiring HIV, are impacted in part by who they see portrayed as at risk in the media. Viewers in these cases are asking themselves “do those portrayed as at risk look like me?” An accurate perception of risk is critical for high-risk populations, who already suffer from a range of health disparities. Yet, to date no study has evaluated the demographic representation of health-related content from social media. The objective of this case study was to apply automated image recognition software to examine the demographic profile of faces in Instagram posts containing the hashtag #HIV (obtained from January 2017 through July 2018) and compare this to the demographic breakdown of those most at risk of a new HIV diagnosis (estimates of incidence of new HIV diagnoses from the 2017 US Centers for Disease Control HIV Surveillance Report). We discovered 26,766 Instagram posts containing #HIV authored in American English with 10,036 (37.5%) containing a detectable human face with a total of 18,227 faces (mean = 1.8, standard deviation [SD] = 1.7). Faces skewed older (47% vs. 11% were 35–39 years old), more female (41% vs. 19%), more white (43% vs. 26%), less black (31% vs 44%), and less Hispanic (13% vs 25%) on Instagram than for new HIV diagnoses. The results were similarly skewed among the subset of #HIV posts mentioning pre-exposure prophylaxis (PrEP). This disparity might lead Instagram users to potentially misjudge their own HIV risk and delay prophylactic behaviors. Social media managers and organic advocates should be encouraged to share images that better reflect at-risk populations so as not to further marginalize these populations and to reduce disparity in risk perception. Replication of our methods for additional diseases, such as cancer, is warranted to discover and address other misrepresentations.

## Introduction

People’s perceptions about health risks are impacted in part by who they see portrayed as at risk, including media portrayals [1]. Specifically, theories in communication and social psychology posit that the perception of prevalence (commonly called the descriptive norm) governs how individuals assess their own risk and adopt prophylactic behaviors [2,3][4,5]. Accordingly if an individual perceives there is low risk among persons they know or see, they likely infer they too are at low risk. In the case of media and disease, these theories assume that when viewers engage with media they ask themselves “do I look like the people who are portrayed as at risk?” [6,7].

It is also well established in the literature on persuasion and health behaviors that people that are perceived as similar to oneself are more influential than people who are perceived as not similar [8]. For instance, through constant social comparison processes, individuals adjust their risk prevalence estimate by changing the reference group to persons who share their traits. Consequently, messages for altering risk reduction behaviors will be more persuasive if delivered by similar others. When the portrayal of those at risk in media is aligned with those of the risk group it is likely to modify perception of risk from those who are indeed most at risk.

These theories garner substantial attention in the evaluation of traditional media (e.g., print or television) [9]. Despite some work on social media [10–12], most analyses are heavily focused on written content in part because early social media was primarily text-based and the most well known analytic strategies rely on text analysis [12–14]. The omission of image analyses focused on social normative theories is critically important as social media is now the dominant source of media consumption among the public [15] and the fastest growing social media channels focus on user generated images and/or video such as Instagram and YouTube. [16].

Furthermore, health disparities are well documented among minority populations, and significantly degrade health outcomes [17]. Perceived lack of representation in visual health communications may further these disparities. This is especially concerning since for many health conditions minority populations are at higher-risk.

### A case study of #HIV on instagram

We selected Instagram as our platform for analysis because it is an exclusively photo and video-sharing social networking service (instagram.com). Instagram has more than 1 billion monthly users who share more than 100 million posts per day [18]. After Facebook, it is the most used social media site and remains the fastest growing, especially among young people [19]. Besides being a primary resource for archiving images, Instagram is also an important medium for sharing health content [20,21]. Instagram users intentionally and unintentionally are defining wellness and health for the larger public [22]. However, the limited public health work using Instagram has largely focused on qualitative methods to examine images [23,24], which do not scale with few exceptions [10–12].

HIV was selected as a vehicle to evaluate our motivating theories for several reasons. First, it is an area of burgeoning social media research and advocacy [25]. Public health has studied how HIV-related organic communications propagate on social media (e.g., how information about preexposure prophylaxis (PrEP) is propagated on Twitter [26]) and leveraged social media to disseminate sexual health information (e.g., efficacy of using Facebook to disseminate sexually transmitted infection prevention messages [27]). Moreover, the US Centers for Disease Control and Prevention (CDC) manages an Instagram account (@ActAgainstAIDS) to promote HIV prevention and control. Concurrently, numerous accounts managed by allied health groups and the lay public may systematically or incidentally promote HIV prevention and control. As of February 2020, there have been more than 600,000 posts that include the hashtag #HIV to Instagram [28].

Second, the tremendous amount of resources applied to HIV prevention and control research means we have significant timely insights into who is at risk of infection to inform our study. For instance, all newly diagnosed HIV infections in the United States are monitored and corresponding details of the case, including the demographics of who are being diagnosed are publicly archived [29]. As a result, it is important to understand how content discussing HIV on Instagram is establishing norms about risk and prevention, namely the norms of the demographics of who is at risk of infection and should engage in prophylactic behaviors. For example, do Instagram images represent the groups most at risk of acquiring a new HIV infection (e.g., black men are at a substantially higher risk of HIV in the US [30]) so when a user views HIV-related content they develop a more accurate perception of their risk and their need to engage in prophylactic behaviors.

Third, HIV in the US disproportionately affects specific populations and numerous reasons mechanisms contribute to disparities in risk, incidence, and screening [31,32]. Many of these disproportionately affected groups, such as black people and men who have sex with men, already face elevated risks of negative healthcare experiences and health outcomes because of underlying dispartities in the US healthcare system [17]Therefore, it is critical to understand if these groups face further challenges due to biases in perceptions in HIV related images on Instagram.

The demographic profile of faces was selected because this represents the emerging focal capabilities of automated image analysis [33]. While automated strategies and their potential application remains debated [34], our contribution is to take an early look at how these methods might be used to benefit public health. Specifically in this study we apply automated image recognition of the demographic profiles of faces in Instagram posts (authored in American English) that contained the hashtag #HIV (and the subset that mention pre-exposure prophylaxis [PREP]) and compare these to the demographics of individuals most at-risk of acquiring a new HIV infection according to estimates of the 2017 US CDC HIV Surveillance Report [29]. In doing so, we aim to answer the question users may pose—“do those portrayed as at risk look like me?”—and start a larger conversation on the study of norm setting in public health related social media images.

## Methods

We assembled a dataset of public Instagram posts from January 2017 and July 2018 using InstaLooter [35]. Posts with the hashtag “#HIV” were collected to capture images that posters self-labeled as primarily related to HIV. Hashtag(s) serve as anchoring devices to label the content of Instagram posts and make it searchable for Instagram users. The posts were then restricted to those authored in American English (i.e., posts with spellings common to the vernacular in the United States) using automated language identification [36] applied to the caption of the post to capture posts that are most likely intended for a US-based audience.

The primary outcomes focused on assessing the demographic attributes of persons included in images with #HIV. *We focused on images that were shared and not the account holder’s profile image*, to measure how specific images about #HIV include persons reflective of the at-risk population. We relied on automated image recognition by Clarifai [37] to evaluate images to discover faces and then estimate its associated demographic profile (age, gender, and race or ethnicity). If more than one face was present in the image, demographics were estimated for each detectable face; if a face was not detectable the person would not be counted (e.g., a back profile of someone looking into the distance). These techniques rely on machine learning, specifically convolutional neural networks (CNN), that are optimized to mirror human judgment, similar to if a researcher reviewed each of the images gathered from Instagram. The CNN ingests an image and returns a probability distribution of the demographic profile of each face. We assigned the demographic characteristic with the highest probability (e.g., we assigned ‘female’ for gender if the model returns a corresponding probability of 70% for female and 30% for male). These methods typically achieve 85% accuracy in face recognition, 88% accuracy in gender recognition, 79% accuracy in racial/ethnic recognition, and 93% accuracy for age estimation when age is grouped [10,38–40]. The automated image recognition analyses can be replicated using the documentation provided by Clarifai [37].

The demographic profiles in the images were normalized to the total number of detectable faces, thereby reflecting the prevalence of each demographic group (age, gender, and race/ethnicity) within #HIV conversations. The resulting representation of demographics in the images in the dataset were described using general descriptive statistics including means, standard deviations, frequencies, and percentages, and compared to the most recent HIV incidence data from the US CDC [29]. Because we analyzed the full dataset of #HIV instagram posts we did not use confidence intervals. In addition to #HIV, we carried out the aforementioned steps on the subset of #HIV posts related to PrEP by restricting the analyses to posts that also contained the keywords “PrEP” or “Truvada”, regardless of capitalization and inclusive of partial words (e.g., “preplife,” “#prep,” “truprep”), anywhere in the caption.

This study was deemed exempt from ethics board review by the University of California San Diego institutional review board because all data was publicly available and the researchers did not interact with the users. However, given the sensitivity of topics discussed and vulnerable groups participating in #HIV conversations, we decided to not include example images after several peer reviewers brought forth concerns about sharing the blurred example images that were included in our initial submission.

## Results

During the 18 month study period, we discovered 26,766 Instagram posts containing #HIV authored in American English. These included 10,036 (37.5%) images with a detectable human face showing a total of 18,227 faces (mean = 1.8, standard deviation [SD] = 1.7). Disparities between the demographic profile of faces in #HIV posts were contrasted with the national HIV at-risk demographic profile according to the estimates in the 2017 US CDC HIV Surveillance Report [29] **(Table 1)**.

**Table 1:** Demographic profiles of #HIV instagram posts vs. at-risk population.

	New Diagnoses^a^	#HIV^b^	PrEP^c^
	%	%	%
**Age**			
<13	0.3	2.8	0.8
13–14	0.1	0.3	0.3
15–19	4.5	0.8	0.6
20–24	16.6	11.7	9.9
25–29	20.1	9.1	12.2
30–34	14.7	1.1	1.3
35–39	11.3	47.0	52.2
40–44	7.8	4.2	3.7
45–49	7.7	9.5	9.9
50–54	7.0	1.2	0.8
55–59	4.9	7.7	6.2
60–64	2.8	2.5	0.9
>65	2.3	2.1	1.1
**Gender**			
Female	19.2	41.3	30.7
Male	80.8	58.7	69.3
**Race or Ethnicity**			
White	26.2	42.6	38.7
Black	43.6	30.8	33.2
Am. Indian / Alaska Native	0.6	0.1	0.2
Asian	2.5	12.9	11.7
Nat. Haw. / Pac. Islander	0.1	0.1	0.1
Hispanic	24.7	13.4	16.2
Multiple Races	2.3	--	--

a. Demographics of newly diagnosed individuals in the US [29]

b. Demographics of faces present in Instagram images referencing #HIV

c. Demographics of faces present in Instagram images referencing PrEP.

The distribution of faces in #HIV images in terms of age was skewed higher than the distribution of newly diagnosed HIV-positive individuals. For example, the majority (47%) of faces represented in #HIV images were 35–39 years old; however, most new HIV diagnoses occur among people that are 25–29 years old.

The distribution of women in #HIV images was skewed higher than the distribution of newly diagnosed HIV-positive individuals. For example, 41% of faces in #HIV images were female, which was more than twice that of new HIV diagnoses (19.2%). Conversely, men were underrepresented in #HIV images, with 58.7% of the faces being male compared to most new HIV diagnoses (80.8%).

The distribution of faces in #HIV images in terms of race and ethnicity skewed towards people who were white, and among minority populations, overrepresented Asians and underrepresented blacks and Hispanics. For example, the majority (42.6%) of faces represented in #HIV images were identified as white; however, most new HIV diagnoses in the US occur among black people (43.6%). Only 30.8% of the faces in #HIV images were identified as black. Similarly, only 13.4% of the faces represented in #HIV images were identified as Hispanic compared to new HIV diagnoses (24.7%). Conversely, Asians were overrepresented representing 12.9% of the faces in #HIV images compared to new HIV diagnoses (2.5%).

Among the subset of #HIV images that also included terms indicative of discussing PrEP, the demographic misrepresentation attenuated only slightly. For instance, the subset of PrEP related facial images also skewed towards people who are older (52.2% were 35–39 years old) and female (30.7%), and less towards those who were black (33.2%) or Hispanic (16.2%).

## Discussion

Our case study found that thousands of Instagram images are contributing to the #HIV discussion; however, the demographic profile of faces in these images does not reflect the demographic profile of the HIV at-risk community. Low risk persons (e.g., white females) are consistently overrepresented and persons with high risk (e.g., black and Hispanic men) are underrepresented. This may be furthering existing health disparities affecting these groups by biasing their perceptions of disease risk. Acting to mitigate this disparity can theoretically make HIV prevention and control advocacy on Instagram more effective. Just as important, our case study is designed to raise questions about why these findings exist for HIV and the larger question about how the field can begin adopting strategies to study norm setting in health-related images on social media.

### Extending theories of media to social media

The importance of social representation, similarity, and norm setting for establishing risk perceptions is well accepted [41,42]. For instance, “targeting” whereby the key demographic and other attributes of individuals appearing in advocacy messages are matched to those of the at-risk audience, is a longstanding hallmark for traditional communication campaigns [43]. For instance, HIV prevention and control campaigns in the US (i.e., “Take Charge. Take the Test.”) have recently featured images of black people because of their high risk of HIV infection [44]. However, thus far, the design of health communication using visual images has been limited to examining the cognitive demand of placement of text in images [45]. Extending concerns about demographic representations to visual social media may be even more critical than their application to traditional media, as the public engages and seeks more health information on social media as compared to traditional media [25, 26]. Understanding existing demographic representation of social media content can inform targeting of public health messaging to ensure that at-risk populations are informed of their relative risk levels, especially those in greatest need or at greatest risk, including future work addressing representation of intersections of demographic groups.

### New methods to study images on social media

Our ability to extend theories of social norms was only possible thanks to the recent advent of automated image analysis tools. While there is tremendous potential in the application of these tools going forward in public health, we would be remiss to not highlight some of the larger debate around these tools.

First, an important consideration is that these tools may be biased and the accuracy is considered preliminary. For example, in general, these tools are more accurate for men versus women and lighter shades of skin versus darker shades of skin. In response to public criticism of their accuracy, several of the automated image analysis tools have been retrained to reduce bias and increase accuracy [34]. Additionally, it is important to note that the concepts of interest within automated image analyses may always be inherently subjective, especially when the concepts of interests are social constructs that only the person in the image can label for themselves (e.g., gender). However, previous work that utilized such a system found it to be reliable in recognizing facial properties such as race [10].

Second, while our conclusions support further research, we urge caution in application of automated image analysis tools. On one hand, computational researchers have outlined the potential for these tools to be misused and result in harm. For example, the potential for structural racism to be reflected in tools that are reliant on demographic inference or authoritarian societies to track members of that society. On the other hand, these tools can be used to potentially reduce disparities in representation in health-related content. For example, our results provide a formative example that helps inject theory and data into the best strategies to help people most at risk of HIV, which also happens to be minorities. Our case study is therefore intended to inspire how health researchers consider how to apply these tools to facilitate new research and intervention trajectories.

Ultimately, the goal of the study and method in which these tools are applied must be carefully evaluated. In our study, our primary goal focused on user perceptions of faces, which can be accurately captured using these tools. Studies that seek to move beyond third party perceptions should consider that race as well as gender are fluid categories in which an individual’s self-identification may be more important than other people’s perceptions. Moreover, the usage of these tools should be guided by the specific context of the study.

### A case for action: Addressing skewed demographics in #HIV posts

Two viable strategies, among many, to address demographic disparities and ameliorate disparities in risk perceptions in Instagram posts could be applied based on our results. First, HIV prevention and control professionals could introduce more demographically representative images by purchasing sponsored Instagram posts or developing public-facing accounts. This approach can use existing staff and resources or automated scheduling of posts using the Instagram API [46]. Second, mirroring strategies applied elsewhere [47], active peer leaders on Instagram could be identified then trained and encouraged to consider demographic sensitivities a part of their messaging. For instance, HIV prevention and control professionals could coordinate marketing efforts around a common branding or theme, much like the #MyTipsForMentalHealth awareness campaign [48], and work with active peer leaders on Instagram to prioritize “at risk” groups in their images. This strategy would foster synergy between professional and lay advocates on social media.

### Extending big media data in HIV prevention and control research

Mining online digital footprints to improve public health surveillance is becoming commonplace in behavioral medicine [14], including breakthroughs in understanding otherwise hidden issues like sexual harassment [49], opioid addiction [50], or sexually transmitted infections [51]. However, HIV researchers have been slow to adopt these strategies. Mining these data (even in a limited way) has already realized several impactful insights for HIV prevention and control [52,53]; including discovering and amplifying the Charlie Sheen effect on HIV testing [54]. Our study adds to this parallel literature by providing a new strategy to mine big media data by adopting automated image analysis to understand norm setting, and is among the earliest systematic studies of HIV on Instagram. In the past, for instance, most studies relied on qualitative content analyses of just a single or a few posts [55,56]. The potential to examine big media data to realize improvements in HIV prevention and control is high, and our study helps identify new directions for this agenda. Moreover, there are several potential avenues for exploration around our simple case study. For instance, researchers might contrast the demographic profile of faces in posts with hashtags about living with HIV (e.g., against the known prevalence of HIV to bring visibility to individuals who are already living with HIV).

## Limitations

Not everyone at risk uses social media; however, Instagram’s demographics align with the target audience of reaching young persons who are at greater risk of acquiring HIV [29]. For example, Instagram is the most popular among young adults (18–29 years old) and people who identify as minorities, especially black or Hispanic [16]. Since we only used public images, we did not capture how HIV prevention and control messages are being broadcast in private networks. Second, our study did not examine underlying factors that could be contributing to the skewed demographics. Although our work identified skewed demographics and suggested a way to fix it, we acknowledge that future work should focus on factors, such as existing social and cultural norms, that could be contributing to this disparity to address the root cause. Third, we relied on usage of English language as a proxy for location. Unlike Twitter, Instagram does not geo-tag posts. Last, at present the technologies around automated image analyses misrepresent multiethnic and non-binary gendered persons. However, we use these technologies to set an early baseline for how to consider demographic norms and biases in health-related social media. In the future as methods for automated image analyses improve it may be possible to even study potential multiethnic or non-binary faces. As algorithmic identification of these identities become available it may help improve our understanding of norm setting on social media or perpetuate stereotypes that might undermine these communities.

## Conclusions

Our case study of demographic profiles on Instagram among #HIV posts charts the course for future research to track and respond to disparities between at-risk population trends and their presentation on social media. There is tremendous potential as automated image recognition methods improve and this underappreciated issue comes to light to engender changes that can impact population-level HIV prevention and control. These changes can be further amplified by replicating our strategy for other health issues and on other social media platforms with visual content.
